# The Greening of Pesticide–Environment Interactions: Some Personal Observations

**DOI:** 10.1289/ehp.1104405

**Published:** 2012-01-18

**Authors:** John E. Casida

**Affiliations:** 1 Environmental Chemistry and Toxicology Laboratory, Department of Environmental Science, Policy and Management, and; 2 Department of Nutritional Science and Toxicology, University of California, Berkeley, California, USA

**Keywords:** ecochemistry, ecotoxicology, metabolism, pesticide, photochemistry

## Abstract

Background: Pesticide–environment interactions are bidirectional. The environment alters pesticides by metabolism and photodegradation, and pesticides in turn change the environment through nontarget or secondary effects.

Objectives: Approximately 900 currently used commercial pesticides of widely diverse structures act by nearly a hundred mechanisms to control insects, weeds, and fungi, usually with minimal disruption of nature’s equilibrium. Here I consider some aspects of the discovery, development, and use of ecofriendly or green pesticides (i.e., pesticides that are safe, effective, and biodegradable with minimal adverse secondary effects on the environment). Emphasis is given to research in my laboratory.

Discussion: The need for understanding and improving pesticide–environment interactions began with production of the first major insecticide approximately 150 years ago: The arsenical poison Paris Green was green in color but definitely not ecofriendly. Development and use of other pesticides has led to a variety of problems. Topics considered here include the need for high purity [e.g., hexachlorocyclohexane and polychloroborane isomers and 2,4,5-trichlorophenoxyacetic acid (2,4,5-T)], environmental degradation and the bioactivity of resulting photoproducts and metabolites, pesticide photochemistry (including the use of structural optimization, photostabilizers, and photosensitizers to achieve suitable persistence), the presence of multiple active ingredients in botanical insecticides, the need to consider compounds with common mechanisms of action, issues related to primary and secondary targets, and chemically induced or genetically modified changes in plant biochemistry. Many insecticides are bird, fish, and honeybee toxicants, whereas herbicides and fungicides pose fewer environmental problems.

Conclusion: Six factors have contributed to the greening of pesticide–environment interactions: advances in pesticide chemistry and toxicology, banning of many chlorinated hydrocarbons, the development of new biochemical targets, increased reliance on genetically modified crops that reduce the amount and variety of pesticides applied, emphasis on biodegradability and environmental protection, and integrated pest- and pesticide-management systems.

Commercially available pesticides currently include approximately 900 structurally diverse compounds (Tomlin 2009) that act by nearly a hundred mechanisms to control insects, weeds, and fungi (Casida 2009b). These pesticides meet the goals of green chemistry (i.e., they are safe, effective, and biodegradable with minimal environmental disruption) to varying degrees. In this commentary, I will review progress in the greening of pesticide–environment interactions, with emphasis on my own observations and research over six decades (Casida 2009a, 2010a, 2010b) concerning the chemistry and toxicology of pesticides in environmental systems [see Supplemental Material, Figure 1 (http://dx.doi.org/10.1289/ehp.1104405)].

**Figure 1:**
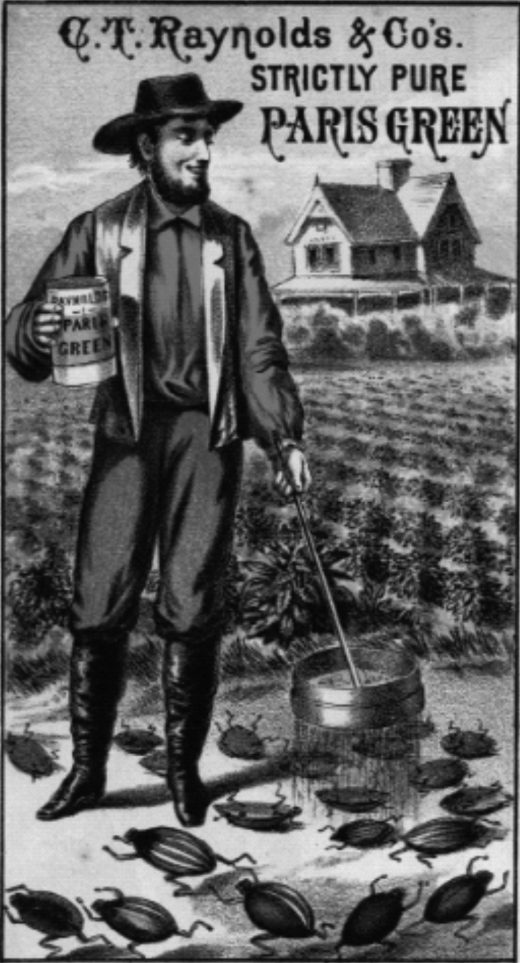
Paris Green, the first green pesticide, was green in color only (definitely not ecofriendly). Label from 1867 package. Reproduced with permission from Getty Images.

The meaning of a “green pesticide” has changed drastically in the last century and a half. Paris Green, the common name for cupric acetoarsenite, is an emerald-green powder containing 43% arsenic and was used from 1865 until the 1940s. It effectively controlled the Colorado potato beetle (Figure 1), chewing pests of cotton and many other crops, and mosquito larvae, with sustained U.S. use levels of about 4,000,000 lb/year (Shepard 1939). Other inorganic toxicants based on arsenic, copper, lead, mercury, sulfur, fluorine, and other compounds—supplemented with the botanicals nicotine, pyrethrum, and rotenone—were also part of the insecticide armamentarium. These compounds provided partial to adequate control for many major pests, but they were far from ideal for environmental safety.

Synthetic organic insecticides introduced in the 1940s and 1950s were far more effective than Paris Green and other early pesticides. DDT (dichlorodiphenyltrichloroethane), chlorinated benzene, chlorinated camphene, and the chlorinated cyclodienes were remarkably successful but introduced new problems of bird, fish, and honeybee toxicity and bioaccumulation through food webs (Stephenson and Soloman 2007; Ware and Whitacre 2004). Paul Müller was presented the Nobel Prize in Physiology and Medicine in 1948 for discovering DDT and its effectiveness in controlling insect-vectored human diseases (Dunlap 1981; Metcalf 1973; Müller 1959), but Rachel Carson was awarded the U.S. Presidential Medal of Freedom posthumously in 1980 for her book *Silent Spring*, which pleaded for banning this insecticide because of its effects on health and the environment (Carson 1962) [see Supplemental Material, Figure 2 (http://dx.doi.org/10.1289/ehp.1104405)]. DDT was highly restricted or banned in 1973 after 4–6 billion pounds had been used. Views on pesticide use and safety continue to differ between food and agricultural producers on one side and environmentalists and health officials on the other. Efforts to understand and cope with these problems were initiated by insect toxicologists who started or revitalized the fields of ecochemistry and ecotoxicology (Felsot 1985). A broad range of information is involved in estimating the environmental impact of specific pesticides (Kovach et al. 1992).

**Figure 2:**
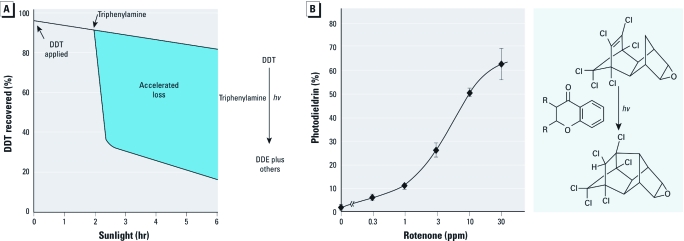
Photosensitizers accelerate insecticide residue loss on bean foliage exposed to sunlight as illustrated for (*A*) DDT (25 ppm) containing triphenylamine (50 ppm; time dependence) and (*B*) dieldrin (10 ppm) containing rotenone (concentration dependence, 1‑hr exposure; error bars are SDs; photosensitization was due to chromanone moiety). *hv*, light energy.

## Ecochemistry

*Impurities and adjuvants.* In contrast to DDT, which is easy to prepare with high purity, insecticides obtained by chlorination of benzene (hexachlorocyclohexane) and camphene (toxaphene) are used as isomer mixtures with 12% active γ-hexachlorocyclohexane (lindane) (Brooks 1977) and 0.2–2% octachlorobornane (A-2), respectively [see Supplemental Material, Figure 3 (http://dx.doi.org/10.1289/ehp.1104405)] (Casida et al. 1974; Saleh et al. 1977; Turner et al. 1975, 1977). Much of the adverse chronic toxicology of technical hexachlorocyclohexane in mammals is probably due to the 5–14% β isomer, which is stored for prolonged periods in fat (Smith 1991). Technical toxaphene consists of several hundred hepta-, octa-, and nonachlorobornanes and related compounds, most but not all of which have been shown to be readily biodegradable based on studies of enzyme, organismal, and environmental fate of the commercial mixture and individual congeners (Maruya et al. 2005; Saleh et al. 1977; Vetter and Oehme 2000). Perhaps the most serious impurity problem was that of 2,3,7,8-tetrachlorodibenzo-*p*-dioxin (TCDD), an impurity in the now-banned herbicide 2,4,5-T (2,4,5-trichlorophenoxyacetic acid; see Supplemental Material, Figure 4) with an oral LD_50_ (median lethal dose) that ranges from 0.6–2.1 μg/kg for guinea pigs to about 1,100–5,000 μg/kg for hamsters (Institute of Medicine 1994). Intraperitoneal administration of [^3^H]TCDD to mice revealed little or no metabolism, an exceptionally long persistence, and high localization in the hepatic endoplasmic reticulum (Vinopal and Casida 1973). Although these examples may be extreme, they highlight the need for high-purity pesticide products.

**Figure 3:**
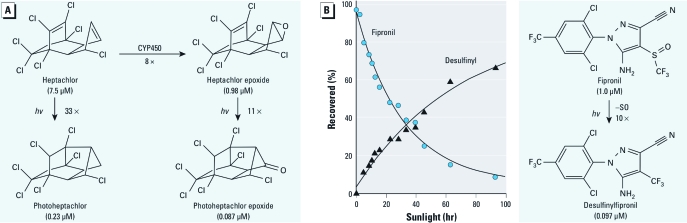
Toxic photoproducts formed during insecticide photodecomposition on bean foliage illustrated by (*A*) heptachlor and (*B*) fipronil. The graph is adapted from Hainzl and Casida (1996). Potencies (micromolar IC_50_ values) are for the γ*-*aminobutyric acid*_A_*receptor noncompetitive blocker site of mouse or rat brain membranes (Hainzl and Casida 1996; Lawrence and Casida 1984). *hv*, light energy.

**Figure 4:**
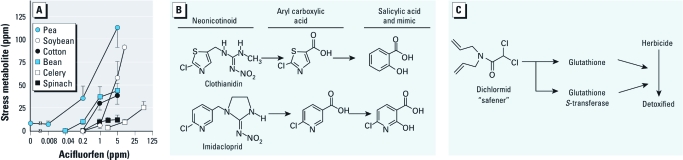
Pesticide-induced changes in plant biochemistry. (*A*) Secondary metabolites induced in six crops by acifluorfen with 48-hr sunlight exposure; pisatin in peas; glyceollins in soybeans; hemigossypol in cotton; phaseolin in beans; xanthotoxin in celery; and *N*-feruloyl-3-methoxytyramine in spinach. Error bars are SDs. Adapted from Kömives and Casida 1983. (*B*) Neonicotinoid insecticides induce salicylate-associated responses in plants. (*C*) Safener induces herbicide-detoxifying enzyme and cofactor in corn but not in weeds.

Solvents, emulsifiers, and a variety of other ingredients, most of which are classified as inert, are added to pesticides to maximize their effectiveness. Although these additives are carefully optimized, they sometimes create unanticipated problems. For example, a reaction between the organophosphorus (OP) insecticide dimethoate and a formulation solvent (2-methoxyethanol) greatly increases dimethoate’s mammalian toxicity without substantially affecting its insecticidal activity (Casida and Sanderson 1961), and a formulation ingredient (polyethoxylated tallowamine) for the active agent in the OP herbicide glyphosate induces apoptosis and necrosis in several human cell types (Benachour and Séralini 2009). These observations are relevant to both human health and environmental health.

*Metabolism.* Pesticide manufacturers must thoroughly characterize degradation by metabolism and photodecomposition to meet requirements for registration and establish residue tolerances, and this information is generally available in the public domain. Pesticides typically yield 10–100 metabolites and photoproducts with varied types and degrees of bioactivity, as illustrated by DDT [see Supplemental Material, Figure 5 (http://dx.doi.org/10.1289/ehp.1104405)]. For example, the dichloroethylene DDE (dichlorodiphenyldichloroethylene), a metabolite of DDT in insects and mammals, is a major persisting environmental pollutant (Smith 1991). *Drosophila* convert DDT to the noninsecticidal but miticidal dicofol (Tsukamoto 1959). DDA, the acetic acid derivative of DDT, is a urinary biomarker of human DDT exposure and also has herbicidal activity (Casida and Allen 1951). Finally, the dichloroethane reductive dechlorination product DDD was detected in Clear Lake, California, ecosystems 7 years after DDD use was discontinued. DDD in the lake appeared to originate from algal metabolism of DDT applied in surrounding agricultural areas (Miskus et al. 1965).

Metabolic activation and detoxification of sulfur-containing OP insecticides is even more complex. Metabolism of profenofos enantiomers yields a mixture of toxicants that includes direct-acting and cytochrome P450 (CYP450)-activated inhibitors of acetylcholinesterase (AChE) and other serine hydrolases (Glickman et al. 1984; Wing et al. 1983) [see Supplemental Material, Figure 6A (http://dx.doi.org/10.1289/ehp.1104405)], which may contribute to its lack of cross-resistance with many other OP insecticides. The OP acephate is bioactivated by deacetylation to methamidophos followed by *S*- or *N*-oxidation to the actual esterase inhibitor (see Supplemental Material, Figure 6B) (Mahajna and Casida 1998). However, the final bioactivated phosphorothiolate can subsequently block the amidase activation step and thus inhibit further bioactivation, which probably contributes to the relatively low mammalian toxicity of acephate (Mahajna et al. 1997).

*Photochemistry.* Pesticides must persist on crops long enough to ensure effectiveness but without causing food-residue problems. The appropriate duration of persistence is normally achieved by structural modifications that improve stability in light without compromising biodegradability. For example, the pyrethroid chrysanthemate and neonicotinoid nitromethylene compounds require photostabilization to be useful in agriculture (Chen and Casida 1969; Dureja et al. 1984; Holmstead et al. 1977; Kleier et al. 1985; Ruzo et al. 1982). The discovery by Farkas et al. (1959) that the insecticidal activity of a pyrethroid was retained when the chrysanthemate dimethylvinyl substituent was replaced with a dichlorovinyl moiety ultimately led to the independent development of the potent photostabilized but still biodegradable pyrethroids by Elliott and colleagues (Casida 2010b) [see Supplemental Material, Figure 7A (http://dx.doi.org/10.1289/ehp.1104405)]. Nithiazine, the primary lead compound for the neonicotinoids (i.e., the discovery from which other neonicotinoids were developed) (Soloway et al. 1979), had a photolabile nitromethylene substituent (Kleier et al. 1985). Its potency was greatly increased with the addition of a chloropyridinylmethyl substituent (prototype) and was subsequently photostabilized with the nitroimine equivalent imidacloprid, currently the most important of all insecticides (Kagabu 2003) (see Supplemental Material, Figure 7B).

Degradation of pesticide residues on leaf surfaces may be enhanced by adding photosensitizers such as triphenylamine to DDT (Figure 2A) or rotenone to dieldrin (Figure 2B), but this results in the formation of bioactive and persistent photoproducts (Ivie and Casida 1970, 1971; Lawrence and Casida 1984). Furthermore, photosensitizers such as rotenone decompose (Cheng et al. 1972) and may have to be reapplied. Pesticides can also serve as photostabilizers; for example, the herbicide trifluralin and other dinitroanilines are quite effective experimental additives when used in pyrethroids for photostabilization (Dureja et al 1984).

Environmentally generated pesticide photoproducts are relevant to efficacy and safety evaluations. Some insecticides are photoactivated to compounds with increased potency as toxicants or receptor inhibitors. For example, the poorly active *Z*-isomer of an oxime ether pyrethroid may be photoactivated to the highly effective *E*-isomer (Brown et al. 1983) [see Supplemental Material, Figure 8 (http://dx.doi.org/10.1289/ehp.1104405)]. The chlorinated cyclodienes generate several toxic photoproducts and metabolites, including photoheptachlor (generated directly from heptachlor) and photoheptachlor epoxide (generated from the CYP450 metabolite heptachlor epoxide) (Figure 3A) (Ivie et al. 1972; Lawrence and Casida 1984). Photochemical desulfinylation of the phenylpyrazole insecticide fipronil produces residues that have equal or greater potency but much greater persistence than the parent compound (Figure 3B) (Hainzl and Casida 1996; Hainzl et al. 1998), which must be considered in approved tolerances and uses for fipronil (Tomlin 2009).

## Ecotoxicology

*Botanical insecticides.* For centuries botanicals have been a principal source of insecticides and insecticidal prototypes for structural optimization. The search for new sources continues, sometimes with surprising results. For example, *Drosophila* bioassays of parsnips unexpectedly revealed a new botanical insecticidal and synergistic natural product identified as myristicin (Lichtenstein and Casida 1963) that is related to dill apiole and parsley apiole, which were later recognized as acting the same way (de Almeida et al. 2009) [see Supplemental Material, Figure 9A (http://dx.doi.org/10.1289/ehp.1104405)]. Similarly, bioassays for house fly toxicity of extracts from 62 plants from central China used in medical practice led to the isolation and structural assignment of paeonol and jacaranone (Xu et al. 2003); another compound of very high potency was identified as terbufos, an extremely hazardous systemic OP insecticide (rat oral LD_50_ 1.6 mg/kg) (Tomlin 2009) (see Supplemental Material, Figure 9B). Thus, botanical insecticides and herbal medicines can be contaminated with synthetic pesticides during production or harvest, thereby possibly confounding the potency of the natural products.

*Common mechanism of action.* There are many examples of chemically diverse pesticides that act on a common primary molecular target. Consequently, it is important to sum the effects of pesticides that have a common mechanism of action when performing risk assessments [U.S. Environmental Protection Agency (EPA) 2011b] or evaluating environmental toxicology, such as effects on birds and fish exposed to multiple OPs and methylcarbamates (MCs) or on honeybees exposed to multiple neonicotinoids. In addition, common mechanisms of action are of great importance to pesticide management practices designed to avoid or forestall the selection of resistant strains by shifting from pesticides with one target site to pesticides that work through a different target, rather than enhancing cross-resistance by using pesticides that have a common target (Fungicide Resistance Action Committee 2010; Herbicide Resistance Action Committee 2010; Insecticide Resistance Action Committee 2011).

*Secondary targets.* Secondary targets for pesticides (i.e., molecular targets not related to their pesticidal activity) are best understood and perhaps of greatest concern for OPs and MCs (Casida and Quistad 2004, 2005). About 90 commercial insecticides that inhibit AChE as their primary target may act on other serine hydrolases as secondary targets. For example, OP-induced delayed neuropathy, first associated with tri-*o*-cresyl phosphate and then with the insecticide candidate mipafox and the insecticide leptophos, is now known to correlate with or result from inhibition of neuropathy target esterase (NTE) (Johnson and Glynn 2001), which has been identified as a lysophosphatidylcholine hydrolase (Quistad et al. 2003; Vose et al. 2007) [see Supplemental Material, Figure 10A (http://dx.doi.org/10.1289/ehp.1104405)]. In mice, the loss of NTE has linked OP exposure to hyperactivity (Winrow et al. 2003). Although hens are the standard model, other avians, sheep, water buffalo, and a variety of other mammals are all considered to be sensitive to effects on NTE (Ehrich and Jortner 2010; Wijeyesakere and Richardson 2010). OP-induced avian teratogenesis, first observed when pesticides were being injected into hen eggs (Roger et al. 1964), is attributable to inhibition of kynurenine formamidase activity and nicotinamide adenine dinucleotide biosynthesis (Seifert and Casida 1980) (see Supplemental Material, Figure 10B). Diazinon and carbaryl induce micromelia and abnormal feathering in hen eggs (Seifert and Casida 1980), but different skeletal defects have been noted for diazinon in bobwhite quail embryos (Meneely and Wyttenbach 1989). The cannabinoid syndrome from OPs involves inhibition of monoacylglycerol lipase and fatty acid amide hydrolase, elevated levels of the endocannabinoids (2-arachidonoyl glycerol and anandamide), and reduced amounts of arachidonic acid (Nomura and Casida 2011) (see Supplemental Material, Figure 10C), but the relevance to wildlife is unknown.

*Changing plant biochemistry.* Herbicides sometimes induce the synthesis of secondary plant substances in crops. At phytotoxic levels, protox inhibitors such as acifluorfen induce phenylalanine ammonia-lyase, which increases phytoalexins and stress metabolites in plants (e.g., pisatin in pea, glyceollin in soybean, and hemigossypol in cotton) (Kömives and Casida 1983) (Figure 4A). Similarly, application of inducers shortly before harvest might be used to elevate levels of desirable botanical products (Kömives and Casida 1982, 1983). Surprisingly, several neonicotinoid insecticides induce salicylate-associated responses in plants (Ford et al. 2010) (Figure 4B). For example, the chlorothiazolylcarboxylic acid metabolite of chlorothiazolyl neonicotinoids induces synthesis of salicylic acid in *Arabidopsis*. In contrast, imidacloprid is metabolized to a chlorohydroxypyridinylcarboxylic acid, which serves as a highly bioactive salicylic acid mimic (Ford et al. 2010).

Plant biochemistry is also intentionally altered to create herbicide-tolerant crops. This can be done on a temporary basis with a safener, or antidote, that enhances sulfate metabolism and elevates the cofactor and enzyme that detoxify the active form of the herbicide, for example, glutathione and glutathione *S*-transferase to detoxify thiocarbamate sulfoxides (bioactivated thiocarbamate herbicides) in corn but not in weeds (Adams et al. 1983; Casida 1978; Lay et al. 1975) (Figure 4C). In addition, 2-oxothiazolidine-4-carboxylic acid (a precursor of cysteine) may be effective in bioremediation to increase chloroacetanilide herbicide detoxification in poplar (Kömives et al. 2003). On a more long-term basis, this approach involves herbicide-tolerant, genetically modified crops (GMCs), for example, that overexpress a less-sensitive form of the 5-enolpyruvylshikimate 3-phosphate synthase target for glyphosate (Dill 2005). As an alternative, glufosinate-tolerant crops express *N*-acetyltransferase that detoxifies by forming *N*-acetylglufosinate. These GMCs may require only (or primarily) glyphosate or glufosinate for weed control, and thus fewer herbicides or smaller amounts of herbicides are applied (Phipps and Park 2002). However, selection of weed resistance to glyphosate threatens the continued effectiveness of GMCs, and GMC technology has not attained global public acceptance.

## The Greening of Pesticide–Environment Interactions

Pesticides vary widely in environmental toxicity and impact. The most important and best available data on effects of pesticides on nontarget species are from acute and chronic exposure safety evaluations in mammals, with additional information on birds, fish, and honeybees (Table 1).

**Table 1:** Ecotoxicology of some major pesticides.*^a^*

Year intro	LD_50_ (mg/kg)*b*			LC_50_ (ppm)	LD_50_*c*	*t*_½_ (days)
	Pesticide type	Mammal	Bird	Fish	Honeybee	Soil
Insecticides												
Paris green*d*		1867		22				Toxic		High		
DDT		1944		113 to > 1,000		Moderate		0.004–0.009		5		90–10,000
Lindane		1945		59–270		120–130		0.02–0.06		0.01		
Toxaphene*d*		1947		40–112		80–250		< 0.05		22–80		
Endosulfan		1955		70–110		205–1,000		0.002		Low		150–240
Carbaryl		1957		264–710		1,000–3,000		1.3–10		0.18		7–28
Chlorpyrifos		1965		135–2,000		32–490		0.002–0.54		0.36		7–56
Deltamethrin		1974		87 to > 10,000		> 2,250		0.00091–0.0014		0.023		8–28
Diflubenzuron		1975		> 4,640		> 5,000		> 65		> 100		3.2
Methoprene		1975		> 10,000				0.37		> 1,000		10
Abamectin		1985		10–221		85 to > 2,000		0.003–0.01		Toxic		Rapid
Imidacloprid		1991		450		31–152		211–237		High		0.17
Fipronil		1993		95–97		11 to > 2,000		0.085–0.43		High		
Tebufenozide		1994		> 5,000		> 2,150		3–5.7		> 234		7–66
Spinosad		1997		3,783 to > 5,000		> 2,000		3.5–30		0.0029		9–17
Flonicamid		2000		884–1,768		> 2,000		> 100		> 60		1.1
Tolfenpyrad		2002		107–386				0.0029				
Chlorantraniliprole		2006		> 5,000		> 2,250		> 14		> 104		< 60–365
Spirotetramat		2006		> 2,000		> 2,000		2.2–2.5		107		< 1
Pyrifluquinazon		2009		300–2,000		1,360		4.4				
Herbicides												
2,4-D		1942		138–764		472 to > 1,000		> 100		104		< 7
Atrazine		1957		> 1,332–3,992		940–4,273		4.3–76		> 97		16–117
Trifluralin		1961		5,545–6,293		> 2,000		0.088		> 100		25–201
Paraquat		1962		22–157		75–175		26–135		15		< 7
Alachlor		1969		930–1,350		1,536		2.1–5.3		> 94		8–17
Glyphosate		1974		3,530 to > 10,000		> 3,851		97 to > 1,000		100		27–146
Chlorsulfuron		1982		5,545–6,293		> 5,000		> 50 to > 980		> 100		28-42
Glufosinate		1981		200–2,000				710 to > 1,000		> 100		7–20
Mesotrione		2001		> 5,000		> 2,000		> 120		> 11		3–7
Fungicides												
Maneb		1950		> 5,000				1.8		Nontoxic		25
Captan		1952		9,000		2,000 to > 5,000		0.034–0.3		91		1
Benomyl		1970		> 5,000				0.27–4.2		> 50		0.8
Triadimefon		1976		250–1,000		> 2,000		4–10.				6–18
Metalaxyl		1979		633–788		923–1,466		> 100		269		29
Azoxystrobin		1996		> 5,000		> 2,000		0.47–1.6		> 25		70
Abbreviations: intro, introduced; LC_50_, median lethal concentration; *t*_½_, half-life. **a**Data from Tomlin (2009) except as indicated. **b**Acute oral LD_50_ values are for the range of species described in the cited study.** c**LD_50_ data are presented as µg/bee by oral exposure except for benomyl, chlorsulfuron, and spinosad, for which data represent contact exposure. Toxicity levels are given as nontoxic, low, moderate, toxic, and high. **d**Data**for Paris Green and toxaphene from Negherbon (1959).												

*Mammals.* Most acute pesticide toxicity problems in mammals are caused by OPs (analogs of chlorpyrifos) and MCs (analogs of carbaryl). Although several insecticides have oral LD_50_ values < 100 mg/kg, the general trend in the last 15 years has been to develop only compounds with reduced mammalian toxicity. Herbicides (other than paraquat) and fungicides are generally less toxic to mammals than are insecticides. The acute oral LD_50_ values tabulated for laboratory mammals are an indicator, but not always reliable predictor, of acute or chronic toxicity to nontarget mammals.

*Birds.* Acute toxicity trends are generally similar for mammals and birds (Table 1). Chronic toxicity also plays a large role in environmental avian responses. The canary was the classical sentinel of toxic gas in coal mines, a role played by the robin and peregrine falcon with environmental DDT exposure; the insecticides underwent food chain accumulation and biomagnification leading to thinning of egg shells and population declines of birds (Carson 1962). Tolerances for carbofuran were revoked (U.S. EPA 2011a) and use of diazinon on golf courses was cancelled (U.S. EPA 2004), in part because of bird mortality. Other insecticides known to cause bird mortality events include the OPs monocrotophos, dicrotophos, methamidophos, and parathion, and the MC aldicarb (Friend and Franson 1999). Strychnine and 4-aminopyridine used as avicides are not only highly toxic to target birds but also pose secondary hazards to predatory and scavenger animals.

*Fish.* Most neurotoxic insecticides have high to ultrahigh fish toxicity, but this is also the case for some nonneuroactive herbicides (e.g., trifluralin) and fungicides (azoxystrobin, benomyl, and captan) (Table 1). Endosulfan, the last of the major chlorinated cyclodienes, was the cause of one of the worst ecological disasters in history (Greve and Wit 1971) when about 70 lb spilled into the Rhine river, killing millions of fish through much of Germany and into the Netherlands [see Supplemental Material, Figure 11A (http://dx.doi.org/10.1289/ehp.1104405)]. Despite its ultrahigh fish toxicity, endosulfan continues to be used for pest management in some countries. The γ-aminobutyric acid–gated chloride channel is the molecular target of several very potent fish toxins, specifically, endosulfan, lindane, toxaphene, and fipronil (Ratra et al. 2001). Toxicity to fish is also a major limiting factor in the use of pyrethroids, such as fenvalerate, particularly when agriculture and aquaculture are in proximity or intermixed (Haya 1989); however, this risk is minimized by proper application methods and the very low field rates required for pest control (Coats 2008). The search for pyrethroids with reduced fish toxicity led to the discovery of the nonester fenvalerate analogs etofenprox and silafluofen, which resulted in expanded use and improved environmental safety in rice production (Tomlin 2009) (see Supplemental Material, Figure 12).

Another pesticide spill may have been California’s worst inland environmental disaster. A tank car of metam sodium, a soil fungicide, tipped over into the Sacramento River, where it degraded into methyl isothiocyanate (the primary active product) and hydrogen sulfide (Carlock and Dotson 2010; Rubin 2004). Further breakdown probably involved methyldithiocarbamate sulfenic acid as an intermediate (Kim et al. 1994; Lam et al. 1993) [see Supplemental Material, Figure 11B (http://dx.doi.org/10.1289/ehp.1104405)]. Although most of these compounds are water reactive and biodegradable, it took many months for organisms in the exposed area to recover (Carlock and Dotson 2010; Gherman 1997).

Fish kill with a pesticide is sometimes intentional. For example, the biodegradable and photolabile rotenone in the form of derris resin (Cheng et al. 1972; Fukami et al. 1967; Schuler and Casida 2001) was used to remove invasive northern pike and other rough fish (i.e., less desirable fish) before reintroducing trout into Lake Davis in California (California Department of Fish and Game 2004, 2008). Lake Davis was treated with derris in 1997 and again 10 years later in an attempt to suppress or eradicate the rough fish. At one time rotenone was also a candidate anticancer agent (Fang and Casida 1998; Gerhäuser et al. 1995) and a model for Parkinson’s disease (Caboni et al. 2004). The primary target of rotenone is reduced nicotinamide adenine dinucleotide oxidase (Horgan et al. 1968; Schuler and Casida 2001), but rotenone also inhibits induced ornithine decarboxylase activity, which serves as an anticancer model (Fang and Casida 1998; Gerhäuser et al. 1995) [see Supplemental Material, Figure 11C (http://dx.doi.org/10.1289/ehp.1104405)]. From the derris added to Lake Davis, 40 components were identified and their inhibitory activity for NADH oxidase correlated with that for the anticancer model (Fang and Casida 1998).

*Beneficial insects.* Honeybees are generally no more sensitive than other insects to insecticides (Hardstone and Scott 2010). However, honeybee losses pose a major problem for agriculture. Pesticides with an LD_50_ < 1 μg/bee include some insecticidal chlorinated hydrocarbons (e.g., lindane), OPs and MCs (carbaryl and chlorpyrifos), pyrethroids (deltamethrin), neonicotinoids (imidacloprid), and microbials (spinosad), but not any of the herbicides and fungicides listed in Table 1. Currently, pesticide levels are high in North American apiaries (Mullin et al. 2010). It is possible to design analogs with low toxicity for honeybees. For example, parathion is highly toxic to bees, whereas its diisopropyl analog is much less harmful (Camp et al. 1969). Many potential uses of imidacloprid and clothianidin are restricted or banned in France, Germany, and Italy because of high bee toxicity, but other neonicotinoids, such as the cyanoimines thiacloprid and acetamiprid, are less toxic to bees (Iwasa et al. 2004).

Insect pests may be adequately controlled by natural predators and parasites until these enemies are removed by insecticide exposure. Integrated pest management programs were therefore developed to optimize biocontrol agents and minimize insecticide effects on biological control (Huffaker and Messenger 1976). Favored chemicals are those with high selectivity for pests versus predators and parasites, including natural and synthetic insecticides, insect growth regulators, and pheromones.

*Other organisms.* Other organisms may be adversely affected by pesticides. For example, earthworms are very sensitive to benomyl fungicide (Karnak and Hamelink 1982; van Gestel 1992), and frogs as tadpoles are sensitive to the lethal effects of endosulfan (Jones et al. 2009). The toxicity and symptomology of pyrethroids in frogs are similar to those in mammals (Cole and Casida 1983). A large number and great variety of pesticides are reported to have reproductive and endocrine-disrupting effects in mammals and wildlife (Colborn et al. 1993). For example, atrazine at environmentally relevant doses has been reported to induce endocrine disruption and demasculinization in frogs (Hayes et al. 2002), although this controversial finding has not been repeated by other laboratories and is not considered to be relevant in safety evaluation (U.S. EPA 2007).

## Conclusion

There has clearly been a greening of pesticide–environment interactions involving improved pest specificity, less nontarget toxicity, lower persistence, and reduced use rates. These successes were sometimes accompanied by unexpected problems, unanticipated hazards, and even major environmental accidents, most of which were solved or placed in risk perspective by fundamental investigations, including studies from my laboratory. Safety has been substantially increased by integrating information related to pharmacokinetic and pharmacodynamic behaviors and operational factors (targeting and use rates), and as our knowledge continues to improve, we can look forward to even greener pesticide–environment interactions.

## Supplemental Material

(561 KB) PDF
