# Supplementary material for: The Greening of Pesticide–Environment Interactions: Some Personal Observations
Source: Environ Health Perspect. 2012 Jan 18;120(4):487–93. doi: 10.1289/ehp.1104405 (PMC3339468; doi:10.1289/ehp.1104405)
Supplement: (561 KB) PDF [file ehp.1104405.s001.pdf]

SUPPLEMENTAL MATERIAL

The Greening of Pesticide-Environment Interactions: Some Personal Observations

John E. Casida

Table of Contents

Supplemental Figure 1. Pesticide-environment interactions.

Supplemental Figure 2. DDT discoverer Paul Müller and anti-DDT wildlife scientist and *Silent Spring* author Rachel Carson. Photo of Müller by the author in 1958. Photo of Rachel Carson about 1944.

Supplemental Figure 3. Insecticides from chlorination of benzene and camphene consist of polychlorocycloalkane isomer mixtures with the most active isomer as a minor component.

Supplemental Figure 4. 2,4,5-T herbicide is banned due to an "exquisitely toxic" tetrachlorodibenzodioxin (TCDD) impurity.

Supplemental Figure 5. DDT metabolism yields a persistent environmental pollutant (DDE), a miticide (dicofol), a plant growth regulator (DDA) and another insecticide (DDD).

Supplemental Figure 6. Metabolic oxidative activation of phosphorothiolate insecticides profenofos (A) and acephate (B).

Supplemental Figure 7. Photostabilization of a pyrethroid (A) and a neonicotinoid (B) by replacing photolabile substituents.

Supplemental Figure 8. Photoactivation of oxime ether pyrethroid.

Supplemental Figure 9. Botanical insecticides in umbelliferae foods (A) and along with a toxic contaminant in Chinese medicinal plants (B)

Supplemental Figure 10. Secondary targets of organophosphorus insecticides.

Supplemental Figure 11. Causal agents in three cases of massive fish kills by accident [endosulfan (A) and metam sodium (B)] and intent (derris containing rotenone) (C).

Supplemental Figure 12. Fenvalerate and two non-ester analogs of reduced fish toxicity

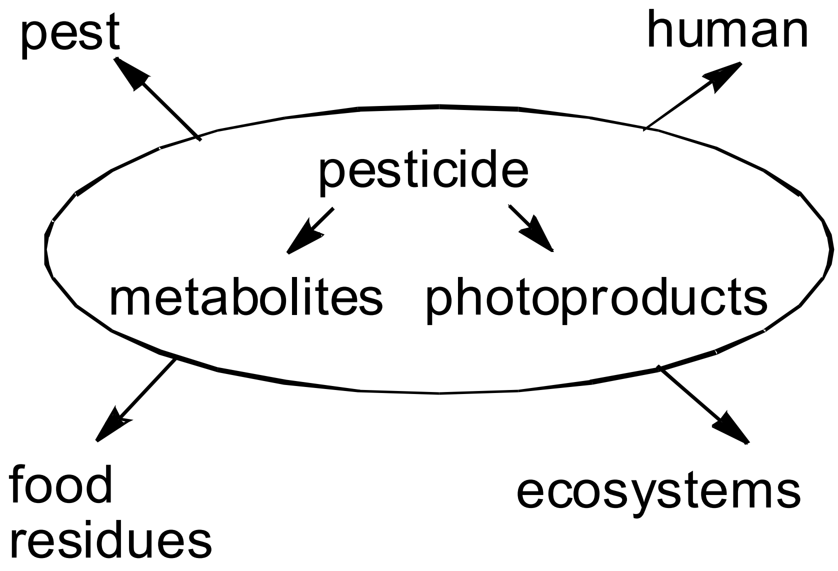

Supplemental Material, Figure 1. Pesticide-environment interactions.

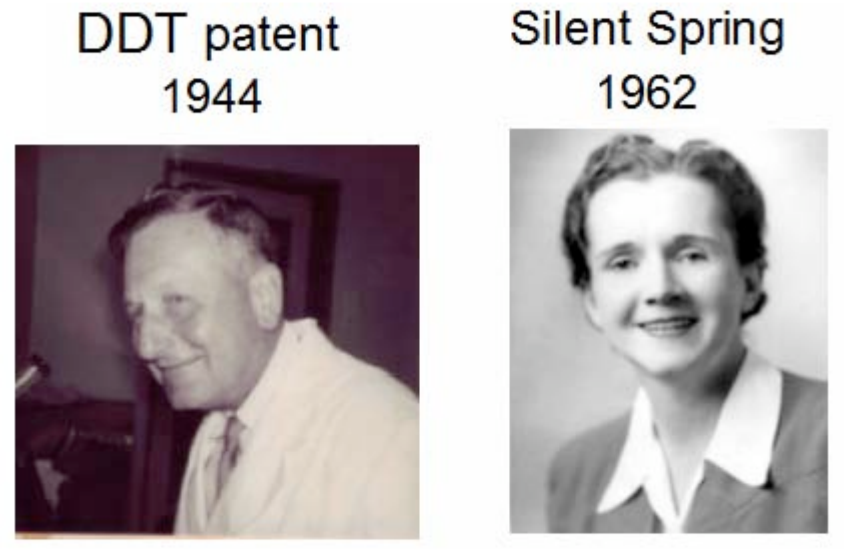

Supplemental Material, Figure 2. DDT discoverer Paul Müller and anti-DDT wildlife scientist and *Silent Spring* author Rachel Carson. Photo of Müller by the author in 1958. Photo of Rachel Carson about 1944.

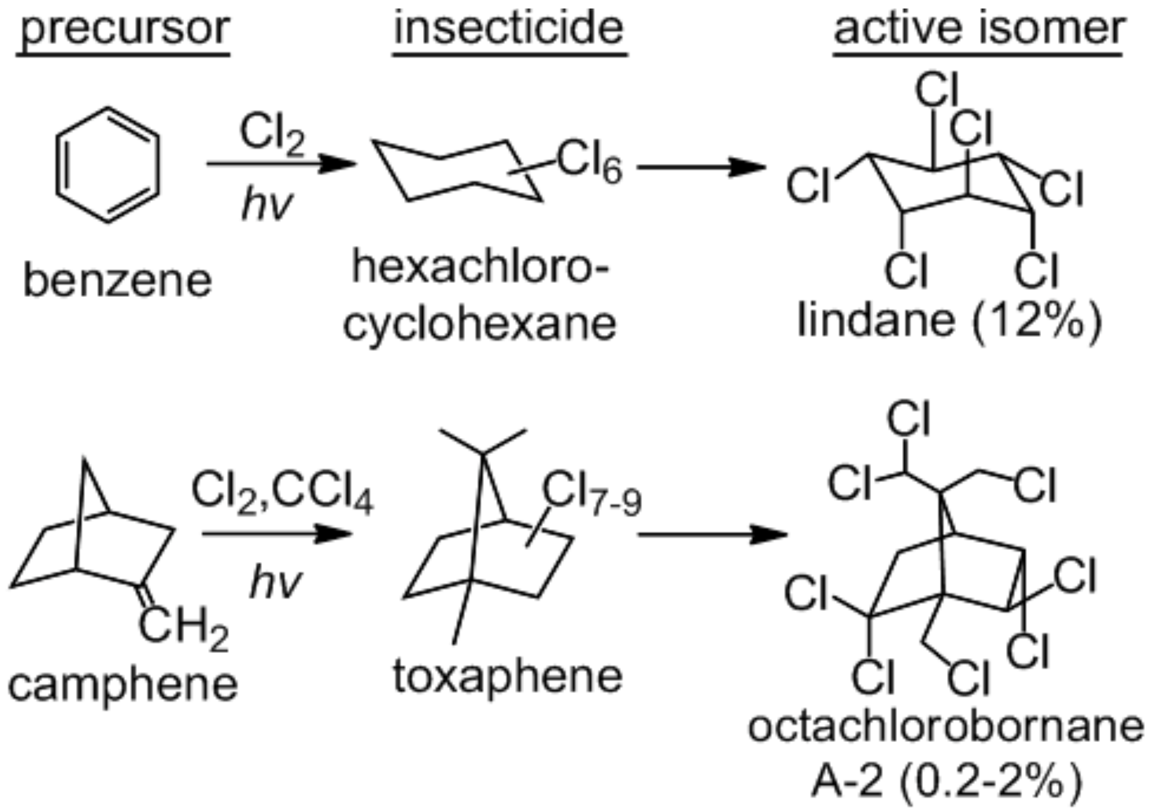

Supplemental Material, Figure 3. Insecticides from chlorination of benzene and camphene consist of polychlorocycloalkane isomer mixtures with the most active isomer as a minor component.

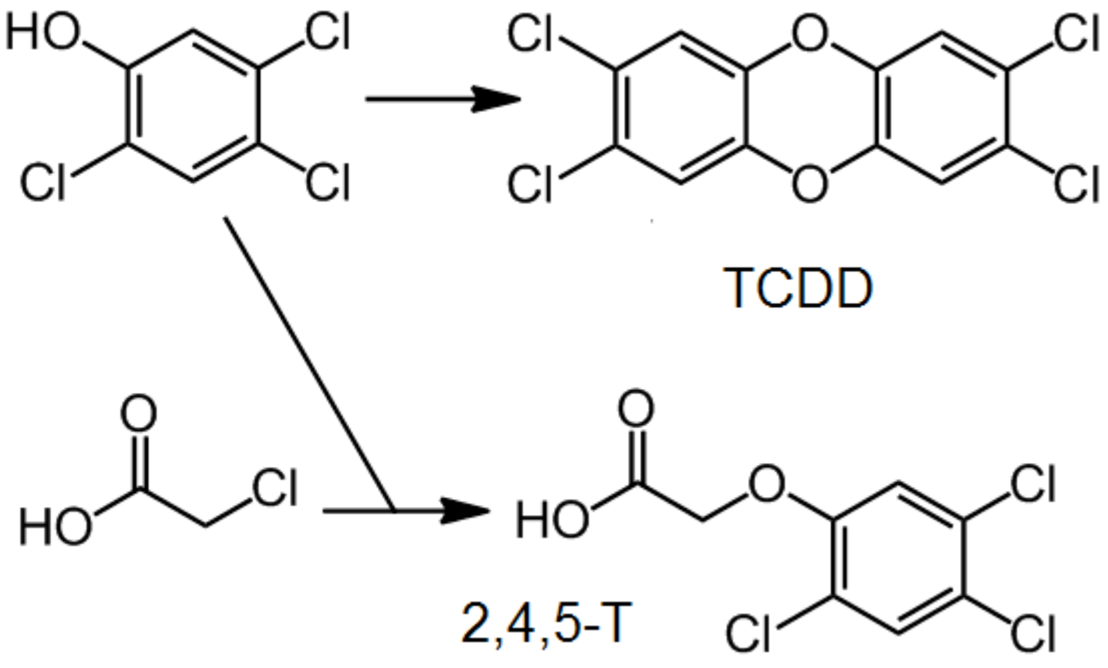

Supplemental Material, Figure 4. 2,4,5-T herbicide is banned due to an "exquisitely toxic" tetrachlorodibenzodioxin (TCDD) impurity.

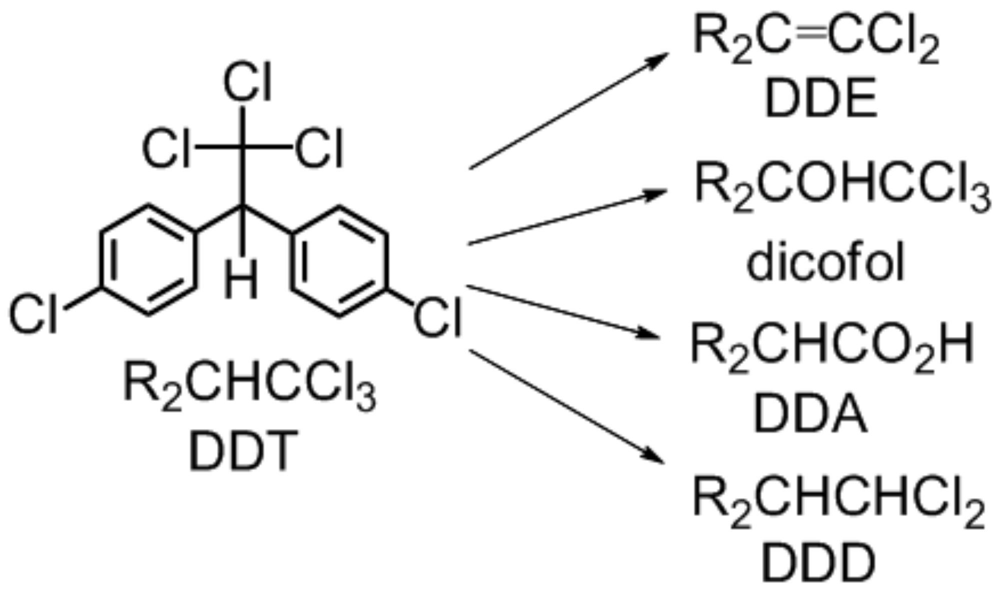

Supplemental Material, Figure 5. DDT metabolism yields a persistent environmental pollutant (DDE), a miticide (dicofol), a plant growth regulator (DDA) and another insecticide (DDD).

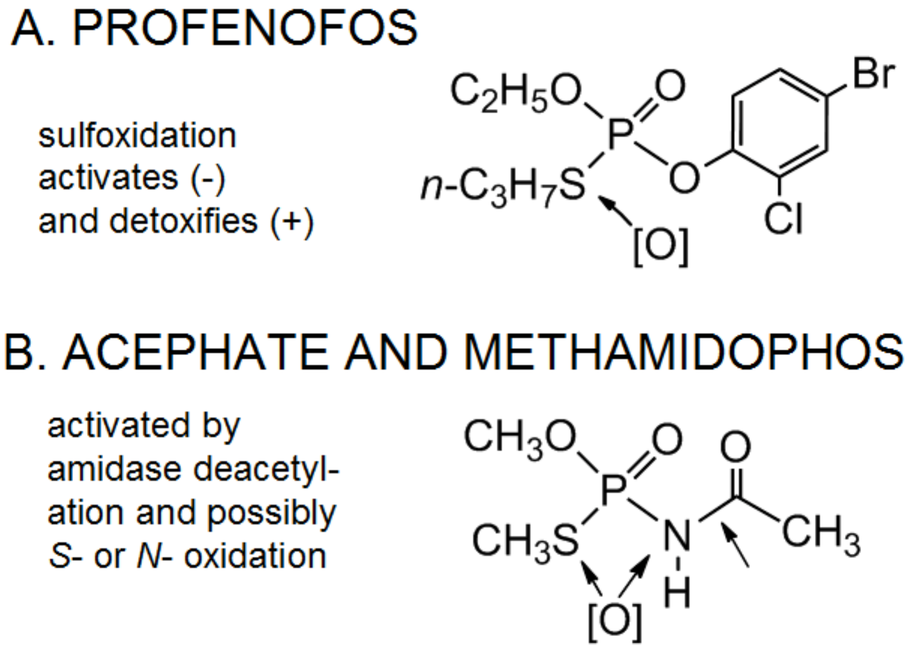

Supplemental Material, Figure 6. Metabolic oxidative activation of phosphorothiolate insecticides profenofos (A) and acephate (B).

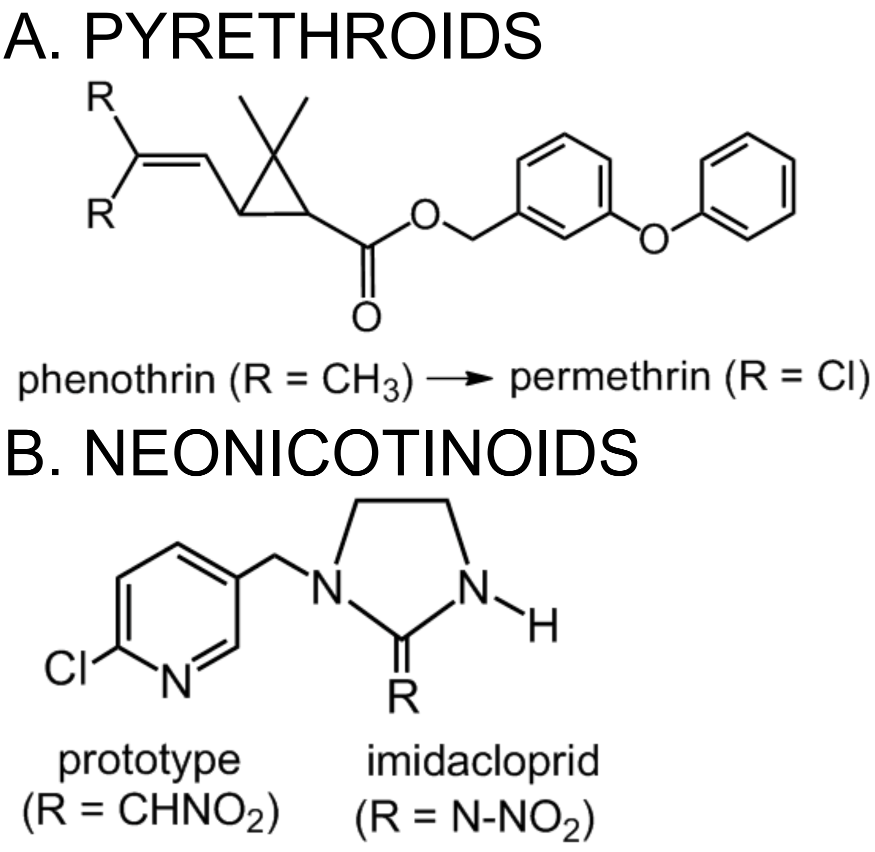

Supplemental Material, Figure 7. Photostabilization of a pyrethroid (A) and a neonicotinoid (B) by replacing photolabile substituents.

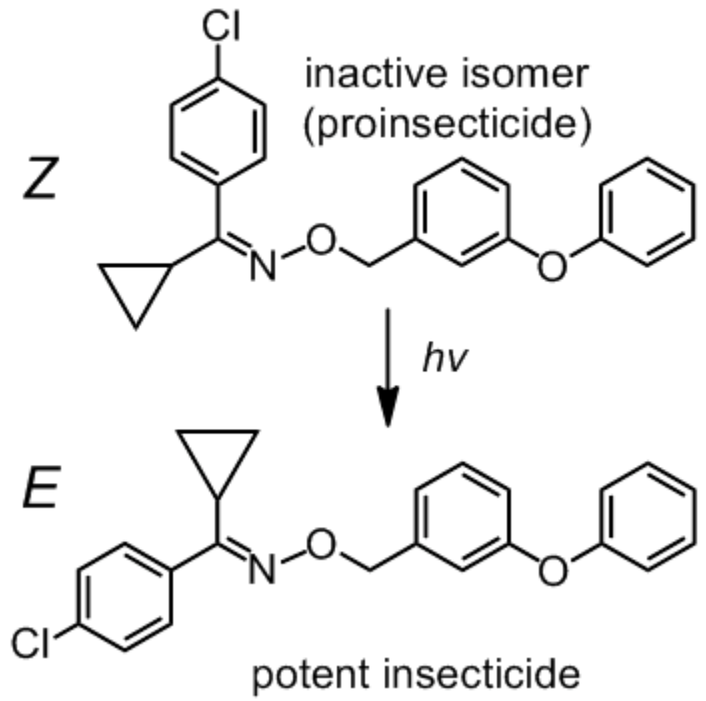

Supplemental Material, Figure 8. Photoactivation of oxime ether pyrethroid.

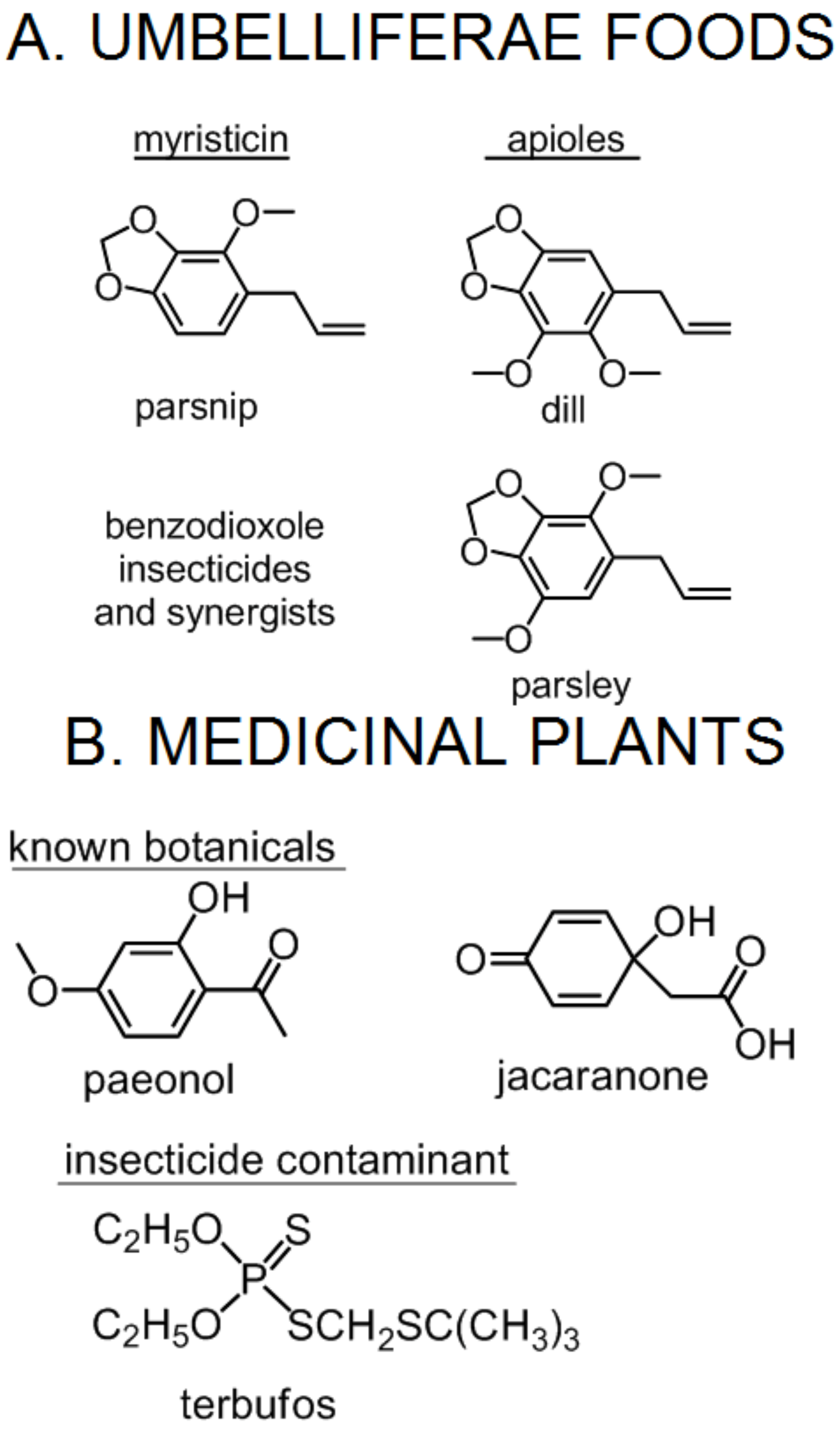

Supplemental Material, Figure 9. Botanical insecticides in umbelliferae foods (A) and along with a toxic contaminant in Chinese medicinal plants (B)

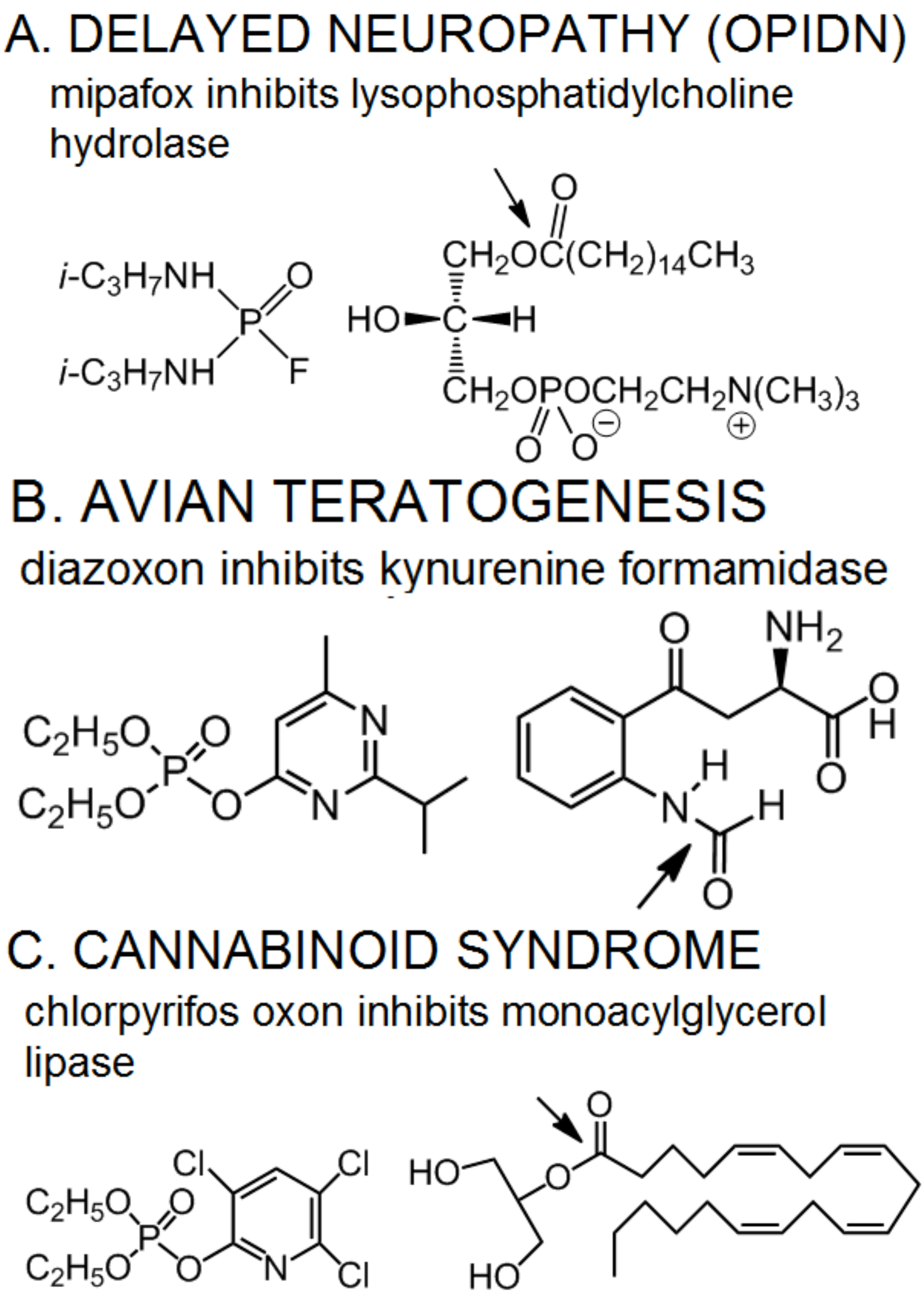

Supplemental Material, Figure 10. Secondary targets of organophosphorus insecticides.

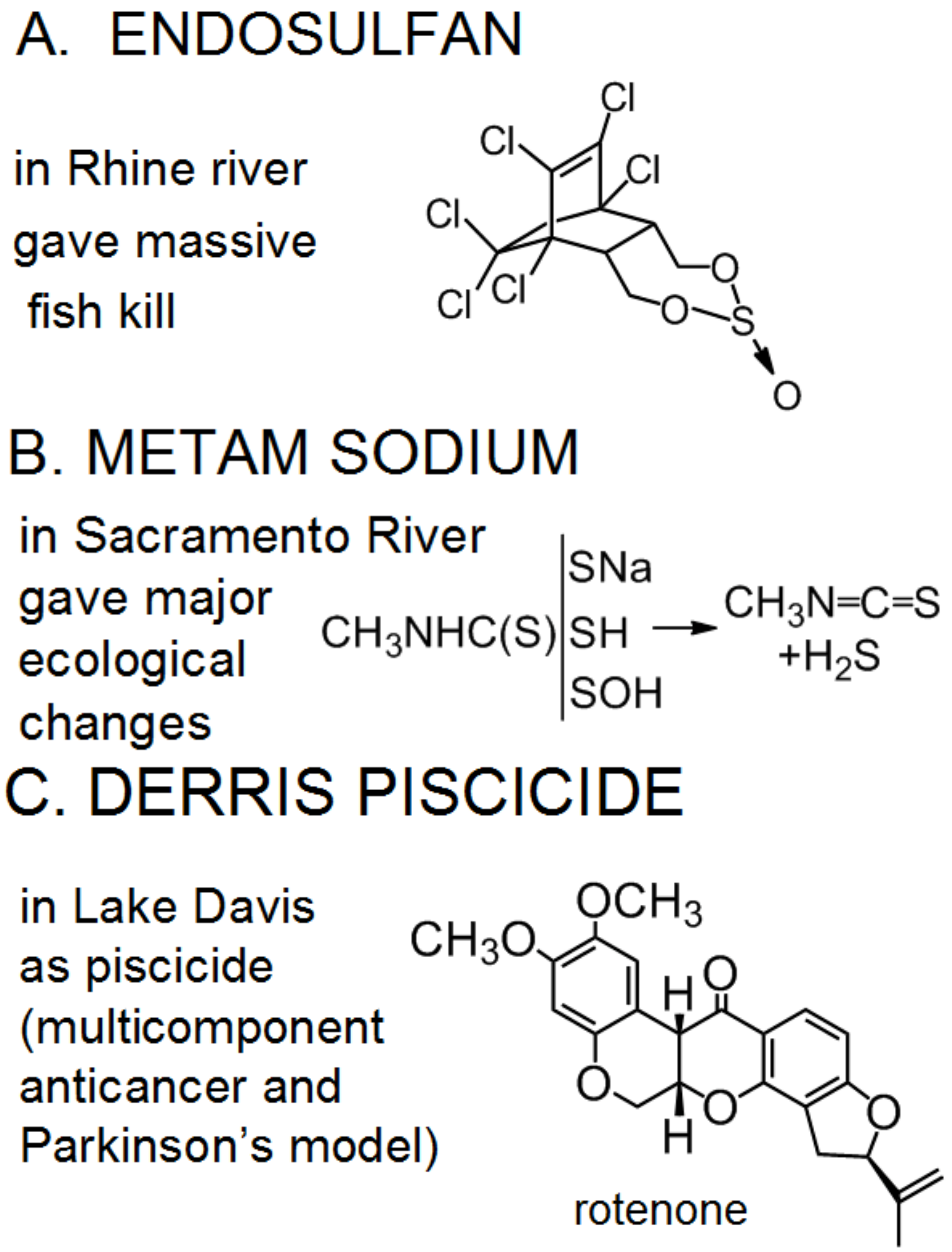

Supplemental Material, Figure 11. Causal agents in three cases of massive fish kills by accident [endosulfan (A) and metam sodium (B)] and intent (derris containing rotenone) (C).

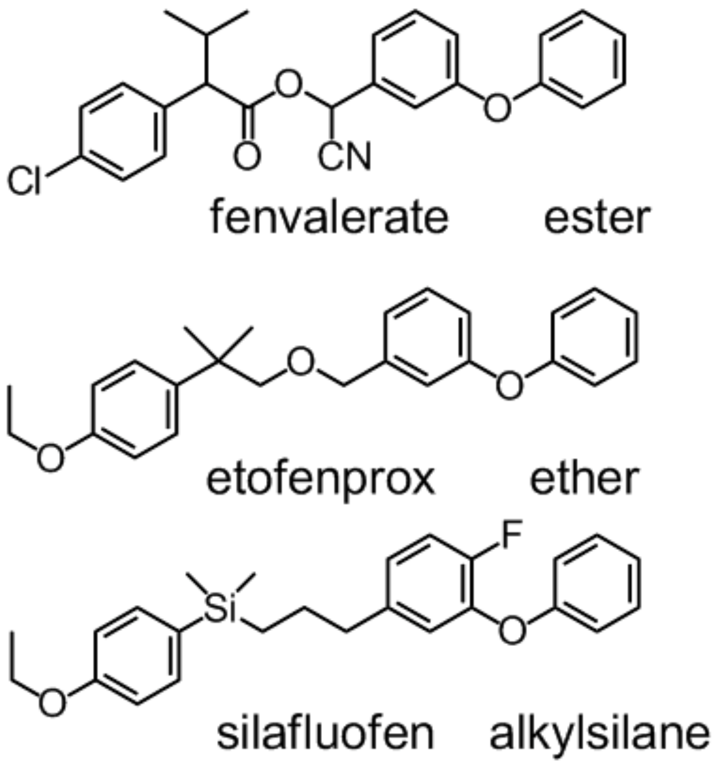

Supplemental Material, Figure 12. Fenvalerate and two non-ester analogs of reduced fish toxicity
